# Electrochemical Impedance Spectroscopy Microsensor Based on Molecularly Imprinted Chitosan Film Grafted on a 4-Aminophenylacetic Acid (CMA) Modified Gold Electrode, for the Sensitive Detection of Glyphosate

**DOI:** 10.3389/fchem.2021.621057

**Published:** 2021-05-07

**Authors:** Fares Zouaoui, Saliha Bourouina-Bacha, Mustapha Bourouina, Albert Alcacer, Joan Bausells, Nicole Jaffrezic-Renault, Nadia Zine, Abdelhamid Errachid

**Affiliations:** ^1^Institut des Sciences Analytiques, Université de Lyon, Villeurbanne, France; ^2^Département de Génie des Procédés, Faculté de Technologie, Université de Bejaia, Bejaia, Algeria; ^3^Departement de Chimie, Faculté des Sciences Exactes, Université de Bejaia, Bejaia, Algeria; ^4^Institute of Microelectronics of Barcelona IMB-CNM-CSIC, Autonomous University of Barcelona, Barcelona, Spain

**Keywords:** microsensor, CMA, MIP, chitosan, Gly, EIS

## Abstract

A novel electrochemical impedance spectroscopy (EIS) microsensor was implemented for the dosage of traces of glyphosate, in real and synthetic water samples. Molecularly imprinted chitosan was covalently immobilized on the surface of the microelectrode previously modified with 4-aminophenylacetic acid (CMA). The characterization of the resulting microelectrodes was carried out by using cyclic voltammetry measurement (CV), scanning electron microscopy (SEM), and electrochemical impedance spectrometry (EIS). EIS responses of the CS-MIPs/CMA/Au microsensor toward GLY was well-proportional to the concentration in the range from 0.31 × 10^−9^ to 50 × 10^−6^ mg/mL indicating a good correlation. The detection limit of GLY was 1 fg/mL (S/N = 3). Moreover, this microsensor showed good reproducibility and repeatability, high selectivity, and can be used for the detection of GLY in river water.

## Introduction

Glyphosate is an herbicide used to destroy what are commonly known as weeds. From the 1970s, the production and use of glyphosate increased steadily around the world. So far, despite the risks involved, this herbicide continues to be widely used (Cuhra et al., 2013). Glyphosate works by interrupting the synthesis of aromatic amino acids essential for the functioning of plants (Lopes et al., 2018). Recently, there has been growing concern about the impact of glyphosate on living things and the environment (Johansson et al., 2018; Seide et al., 2018). Among the many effects of this herbicide are toxicity, changes in the activity of antioxidant enzymes, endocrine disruption, damage to lipids, histological damage, etc. (Lopes et al., 2018; Ren et al., 2018; Lorenz et al., 2019). Glyphosate can be found as a contaminant in soil, plants and food products. GLY has a high solubility in water and its intensive use leads to contamination of surface and ground water (Ruiz de Arcaute et al., 2018). A certain number of approaches have been adopted for the detection of glyphosate in various media such as chromatography, photometry, mass spectrometry, etc. (Clegg et al., 1999). However, these techniques are long, difficult to implement, expensive, and often require qualified personnel. Moreover, their application is limited to the laboratory scale.

The challenge for analytical chemistry is to develop alternative analytical methods with an emphasis on simplicity, efficiency and cost. Molecularly imprinted polymers (MIPs) seem to be a very good alternative for designing analysis tools because of its many properties such as selectivity, robustness, physical and chemical resistance, inertia and ease of synthesis and Low cost (Haupt and Mosbach, 2000, Qiao et al., 2006). MIPs can selectively recognize a molecule or even a family of analogous molecules. Molecular printing consists of creating cavities, which are complementary from the structural and functional point of view of the target molecules within a functional polymer. The synthesis of MIPs is the interaction of the functional monomer with template molecules through covalent, semi-covalent, and non-covalent interactions (Yan and Row, 2006); which leads to the formation of the pre-polymerization complex. After the addition of a crosslinking monomer, a polymer network is formed around the functional monomer/template complex by copolymerization between the functional monomers and the crosslinking monomer, thus allowing the formation of a more resistant and more stable matrix (Wulff et al., 1983). As a last step, cavities complementary to the target molecules with regard to their size and the presence of specific functional groups are obtained after the extraction of the template from the polymer. The porous matrices obtained can be used to specifically recomplex the target molecules present in various media. The selectivity of MIPs is conditioned by the functional monomer involved in the synthesis of molecular printing. The monomer is chosen according to its physicochemical properties and its ability to interact with the template (Pichon and Haupt, 2006).

The many properties of chitosan (CS) allow it to be a very good choice for use in the synthesis of MIPs. It is in particular biocompatible, biodegradable, non-toxic and has good antimicrobial and antioxidant activities (Younes and Rinaudo, 2015). In addition, this material has various physical and chemical characteristics. It is considered to be the second most abundant natural biopolymer after cellulose. Chitosan is a natural cationic polysaccharide (in dilute acid medium) extracted from chitin having as reactive functional groups a primary and secondary hydroxyl group and a primary amino group (Pa and Yu, 2001).

The strong functionality of chitosan through its hydroxyl and primary amine functions gives it a considerable opportunity of chemical modification (Aranaz et al., 2010). These modifications were intended in order to enhance the characteristics and internal morphology of the CS or to give it new properties (Wang et al., 2016). Many studies have looked at the modification of chitosan (Martins et al., 2004; Pereira et al., 2013). Chitosan is biofunctionalized according to a very common technique resulting in the formation of a bond of the primary amine of chitosan with a carboxylic acid (Chung et al., 2002). Nucleophilic reactions on electrophilic carbons such as aldehydes, ketones and carboxylic acids are thus made possible by the non-binding nucleophilic doublet possessed by the primary amine. The use of chitosan in the MIP matrix is increasingly widespread and the number of published works reporting the extent of research in this area is constantly expanding (Karrat et al., 2020; Zouaoui et al., 2020a). Chitosan combined with the MIP technique have been reported for the preparation of the electrochemical sensors for different target molecules including pharmaceutical compounds (Lin et al., 2015; Song et al., 2019), protein (Fatoni et al., 2014; Xia et al., 2016), sweet (Li et al., 2014), phenolic compounds (Deng et al., 2013; Li et al., 2013; Chakroun Galai et al., 2020; Salvo-Comino et al., 2020), organic compounds (Chen et al., 2011), ions (Wu et al., 2020), and pesticides (Zouaoui et al., 2020a).

Several sensors are being developed by electrodeposition of molecularly imprinted chitosan. The application of a negative potential during the electrodeposition of chitosan causes release of hydrogen gas. H_2_ remains trapped in the membrane of the CS which makes it inhomogeneous and affects its sensitivity. The covalent grafting of chitosan allows eliminating these effects and achieving a more stable response (Zouaoui et al., 2020a).

In this work, covalent modifications of the surfaces of gold microelectrodes were explored by the use of a 4-aminophenylacetic acid, more commonly called CMA, to ensure the covalent immobilization of molecularly imprinted chitosan in order to develop microsensors, distinguished to detect traces of glyphosate in water.

The process used to deposit CMA resides in the formation of a diazonium salt cation which makes it possible to form a covalent bond between the acid and the gold surface. The diazonium cation is an unstable species, easily hydrolyzable at room temperature and very reactive (Chira et al., 2017).

The diazonium ion can be prepared by a standard diazotisation procedure, by treatment of aromatic amines with nitrites ${\text{NO}}_{2}^{-}$ in the presence of hydrochloric acid (HCl). After this treatment, the –NH_2_ group of the aniline derivative was converted to a –${\text{N}}_{2}^{+}$ group leads to the formation of an aryl diazonium. The electrochemical reduction of the diazonium cation on a metal cathode by an electron transfer from the surface of the electrode to the diazonium salt generates an intermediate aryl radical after the release of N_2_. The aryl radical attaches to the conductive surface by formation of a covalent bond between the carbon of the aromatic cycle and the metallic element (Corgier et al., 2005).

The carboxylic function of the acid is then available as an accessible surface of the electrode for grafting chitosan. CS is functionalized by the CMA via the formation of an amide bond. The pair 1-ethyl-3- (3-dimethylaminopropyl) carbodiimide and N-hydroxysuccinimide (EDC/NHS) makes possible the preceding amide bond, thanks to its carboxylic acid function. It consists of the formation of an ester intermediate between NHS and the carboxylic function. The intermediate thus formed reacts more easily with the primary amine of chitosan which improves the reaction kinetics. Better adhesion of the chitosan will thus make it possible to obtain a homogeneous functionalized chitosan membrane on the surface of the sensor (Corgier et al., 2005).

## Materials and Methods

### Reagents

Chitosan (CS, degree of deacetylation 80.0–95%), acetic acid (purity 99.7%), methanol (purity 99.9%), 1-ethyl-3-(3-dimethylaminopropyl)carbodiimide (EDC), 4- aminophenylacetic acid (CMA), sodium nitrite (NaNO_2_), hydrochloric acid (37%, HCl), Pure ethanol (99.8%), Sulfuric acid (purity 95%), sodium hydroxide (NaOH) and glyphosate were obtained from Sigma Aldrich, France. N-hydroxysuccinimide (NHS) was purchased from Acros Organics, France. All interferents: gluphosinate-ammonium (GLU), chlorpyrifos (CHL), and phosmet (PHO) were supplied by Sigma Aldrich, France. The experimental runs were performed at room temperature with analytical grade reagents.

### Apparatus

**Figure 1:**
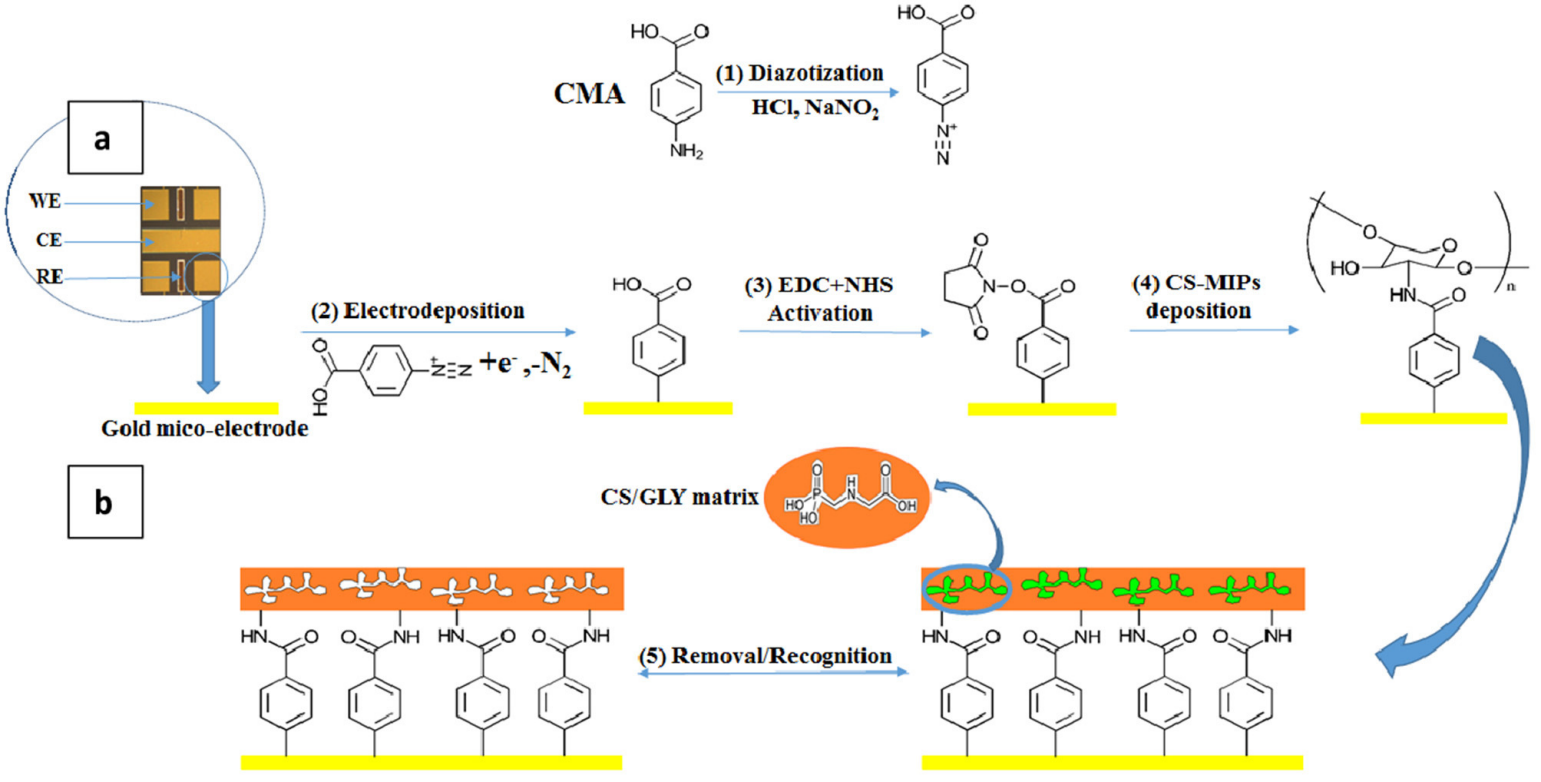
**(A)** Transducer holding an array of four bare-gold working microelectrodes (WE) *s* = 0.64 mm^2^, one counter microelectrode (CE) *s* = 0.13 mm^2^, and two Ag/AgCl reference microelectrodes (RE) *s* = 1.37 mm^2^. **(B)** Preparation of CS-MIPs/CMA/Au and its recognition for GLY.

The data of electrochemistry were obtained through a multichannel potentiostat analyzer (Biologic-EC-Lab VMP3). The tests were performed with a transducer supplied by the National Center for Microelectronics (CNM), CSIC, Barcelona. It's made up of a plate of four bare gold working microelectrodes (WE) (*s* = 0.64 mm^2^), one counter microelectrode (CE) (*s* = 0.13 mm^2^), and two Ag/AgCl reference microelectrodes (RE) (*s* = 1.37 mm^2^) (see transducer in Figure 1). The use of the four working microelectrodes allowed us to go faster in the experimental part. With the multichannel potentiostat it was possible to prepare four microsensors at the same time and to use them simultaneously either for detection, interference studies, regeneration, and analysis of the real sample. With the responses of the four microsensors we made the error bars.

A pH- meter: Mettler Toledo FE20/EL20 was used for the measure of solution pH. A FEI Quanta FEG 250 SEM (University of Lyon 1), France, was used to obtain the Scanning Electron Microscopy (SEM) images.

### CS-MIPs/CMA/Au Sensor Fabrication

#### Diazonium Grafting Onto Gold Microelectrodes

##### Preparation of Diazonium Salt (CMA)

3 mM CMA solution was prepared with 20 mM of HCl (20 mM) and 20 mM of NaNO_2_ (20 mM) in an aqueous solution (Figure 1 step. 1). Then, the mixture was stored at 4 °C until the appearance of a pinkish hue (about 5 min) (El Alami El Hassani et al., 2019).

##### Electrodeposition of CMA

**Figure 2:**
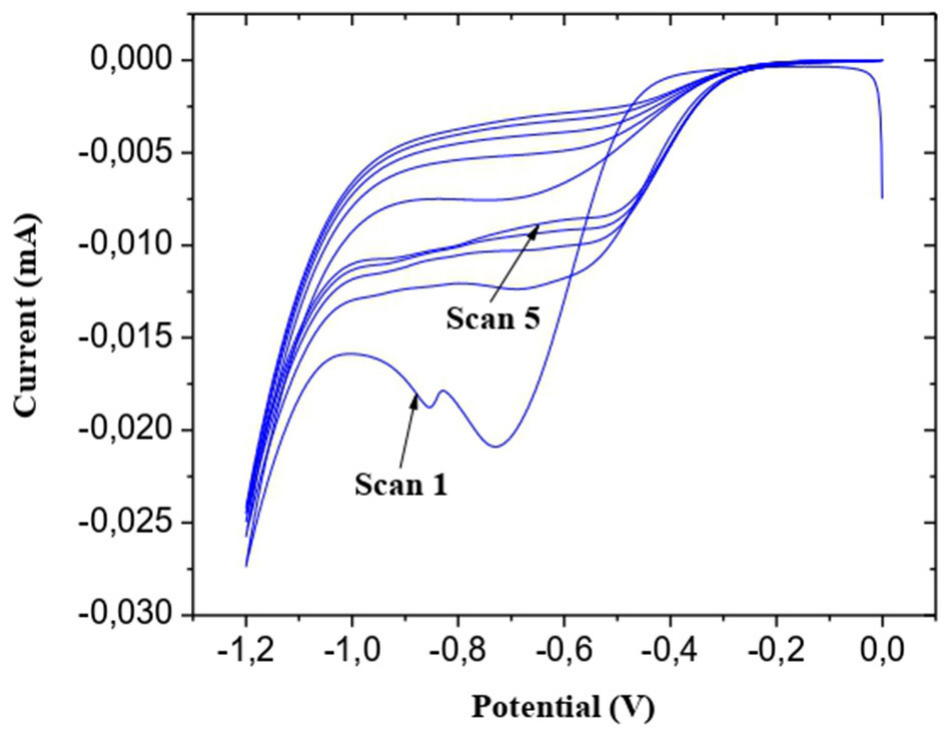
Cyclic voltammogram of electrodeposition of CMA on the microelectrode surface.

The Au microelectrodes were washed beforehand for 10 min with ethanol and deionized water under ultrasound. The final step is their exposure for 30 min to UV/ozone. 5 cyclic voltammetry (CV) scans between −1.2 and 0.0 V at a scanning rate of 50 mV/s were carried out for the electrodeposition of the CMA solution (Figure 1 step. 2). A cathodic peak is observed; the decrease in maximum intensity with each successive sweep (Figure 2) is probably due to the obstruction of the working electrode surface. After electrodeposition, the microelectrodes undergo rinsing with deionized water and drying under a stream of nitrogen.

##### Activation of the Carboxylic Function

Solution of 400 mM of 1-ethyl-3- (3 dimethylaminopropyl) carbodiimide (EDC) and 100 mM of N-Hydroxysuccinimide (NHS) was prepared in absolute ethanol. The microelectrodes functionalized by the CMA were incubated in this solution for 1 h to activate the carboxylic function (Figure 1 step. 3). Then, the devices were carefully rinsed with pure ethanol and dried using nitrogen (El Alami El Hassani et al., 2019).

#### Grafting of CS on Activated CMA

##### Preparation of GLY/CS Mixture

To prepare the CS solution, 1 g of CS powder was dissolved in a volume of 100 mL of acetic acid 0.1 M and ultrasonicated for 6 h at room temperature. 1 mg/mL of GLY was prepared in CS solution and stirring of the suspension for 2 h is followed by an adjustment of the pH to 5 with a solution of ammonia hydroxide. Chitosan used has a medium molecular weight (≈250 kDa). The monomer/template ratio is 1/1, that means that in the mixture consists of one glyphosate molecule for each glycosamine monomer. The molecular weight of chitosan being 250 kDa which correspond to 1,470 glycosamine monomers per polymer chain, the mixture contains one molecule of glyphosate for one glycosamine monomer.

##### CS-MIPs Deposition

GLY/CS mixture was deposited by drop-casting on the functionalized microelectrodes surface for 24 h. The deposition was followed by incubation during 1 h of the GLY-CS/CMA/Au in 0.5 M H_2_SO_4_ as the cross-linker to remove residues of chitosan. Chitosan is soluble in all mineral acids except sulfuric acid. In this case, sulfuric acid is used as a crosslinking agent during the formation of the biopolymer. The deposited CS film remains stable; moreover the reproducibility results confirm this (see section About the Sensor Regeneration and Reproducibility). During the crosslinking, ionic bonds are formed between the sulfate ions of sulfuric acid and the ammonium ions of chitosan. The mechanism of crosslinking of chitosan with sulfuric acid is presented in Supplementary Figure 1.

To eliminate the molecule template, a second incubation of about 30 min was made in acetic acid/methanol solution (1:1, v/v). The incubation time of 30 min is considered to be an optimal time for maximum extraction of GLY from the CS film. Because for an incubation time >30 min, EIS plot and CV voltammogram can be superimposed on those for an extraction time of 30 min. Knowing that initially the GLY (anionic) is adsorbed on active sites of chitosan (cationic biopolymer) by electrostatic interaction at pH = 5, it is desorbed in AcAC/methanol (1:1; v:v) solution at pH <4.5 for the most part. While the chitosan remains adherent and fixed by covalent bonds to the surface of the functionalized microelectrode.

So, a microsensor constructed with CS molecularly imprinted film modified with CMA was applied for a specific recognition of GLY. The process is shown in Figure 1 (step. 4 and 5).

The steps in making the unprinted polymer sensor (CS-NIP/CMA/Au) are identical to those for the CS-MIP/CMA/Au microelectrodes, except that GLY was absent from the entire process. Storage at ambient temperature of the sensors was carried out for further applications.

### Experimental Conditions

A multichannel potentiosat (Biologic-EC-Lab VMP3) was used for the characterization by the techniques of cyclic voltammetry (CV) and electrochemical impedance spectroscopy (EIS) of all the modifications made to the working microelectrodes. All the measurements were performed in 5 mM ferro-ferricyanide ([Fe(CN)_6_]^3−/4−^) and phosphate buffer saline solution (PBS). For the cyclic voltammetric measurements, the potential was scanned between 0 and 0.45 V at a scan rate of 80 mV/s. EIS data are obtained for an initial potential of 0.2 V and a frequency range varying from 300 mHz to 100 kHz. For detection step, after an immersion in aqueous GLY solutions of concentrations varying between 0.31 × 10^−9^ and 50 × 10^−6^ mg/mL for a period of 30 min, CS-MIPs/CMA/Au electrodes were characterized by EIS.

## Results and Discussion

### Characterization of the Surface Modifications

Electrochemical impedance spectroscopy (EIS) has emerged as an alternative to conventional analysis techniques to study the modified surfaces. EIS provides information on the kinetics of molecular phenomena at the interface, as well as on the coverage rates and the effectiveness of surface modifications (Proud and Müller, 1993). By modeling the electrochemical response by equivalent electrical circuits, it is possible to relate the physical and chemical properties of the electrode material to the data from the EIS curves (Janata, 2002).

**Figure 3:**
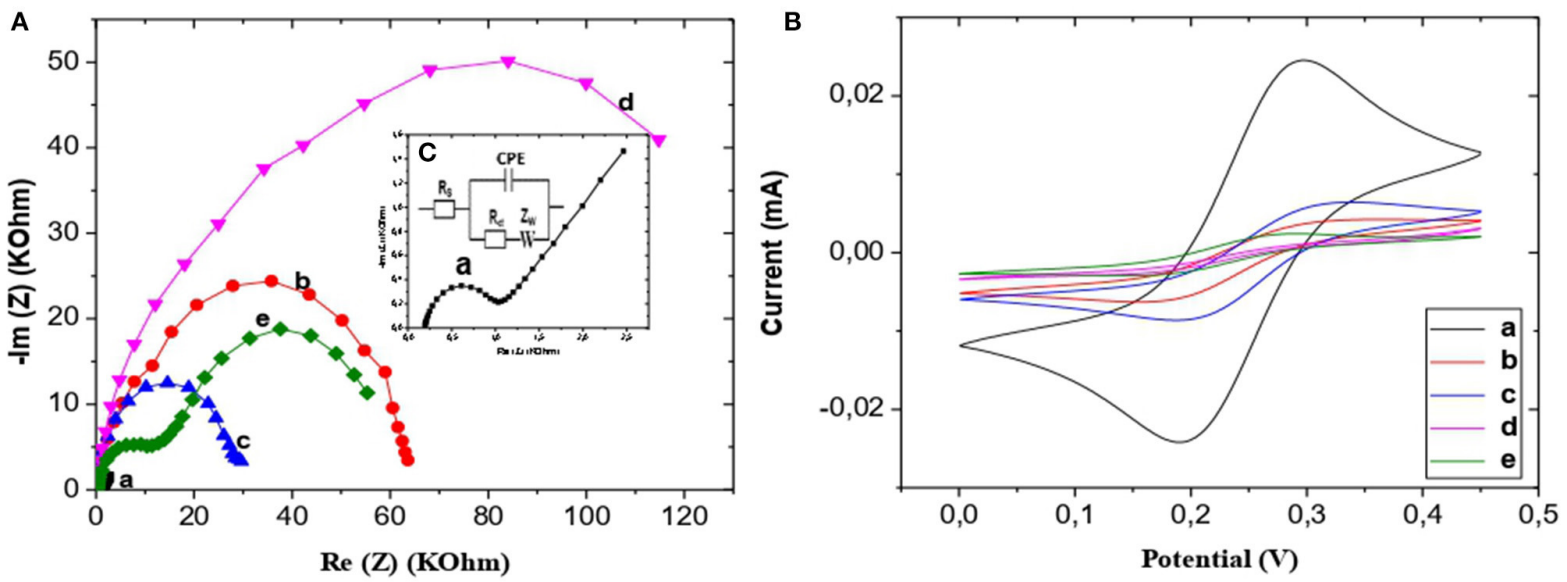
**(A)** EIS of bare Au (a), CS-NIPs/CMA/Au (b), CMA/Au (c),CS-MIPs/CMA/Au before extraction of template (d), CS-MIPs/CMA/Au after extraction of template (e), in 5 mM [Fe(CN)_6_]^3−/4−^ and PBS, initial potential E = 0.2 V. Highest Freq = 100 khz, Lowest Freq = 300 mHz. **(B)** VC of bare Au (a), CS-NIPs/CMA/Au (b),CMA/Au (c),CS-MIPs/CMA/Au before extraction of template (d), CS-MIPs/CMA/Au after extraction of template (e), in 5 mM [Fe(CN)_6_]^3−/4−^ and PBS, from 0 to 0.45 V at a scan rate of 80 mV/s. **(C)** Randles' circuit, where R_s_ is the electrolyte resistance, CPE is the constant phase elements, R_ct_ is the charge transfer resistance and Z_w_ is the Warburg impedance.

The equivalent circuit shown in Figure 3C was used to fit the Nyquist plots a, b, c, and d of Figure 3A, and the equivalent circuit shown in Figure 5B was used to fit the Nyquist plots e of Figure 3A.

From the examination of Figure 3A, a, it emerges that the sensor has a high charge transfer rate for [Fe(CN)6]^3−/4−^ (R_ct_ = 783 Ohm). An obvious increase of the charge transfer resistance value was observed with the electrodeposition of CMA on the gold microelectrode surface (R_ct_ = 26,269 Ohm) (Figure 3A, c). Impedance becomes higher value after the chemical deposition of chitosan (R_ct_ = 95,452 Ohm) (Figure 3A, d).

From the comparison of the diagrams obtained with the NIP film (Figure 3A, b), the MIP film (Figure 3A, d) has a larger Re (Z), and therefore, a greater thickness than that of the NIP film. Indeed, the NIP, the chitosan chains are positioned in a flat manner on the CMA. Logically, the thickness of the NIP membrane equals the width of the glucosamine in the chitosan.

For the MIP, the presence of glyphosate gives a film with curvatures which modifies the morphology of the film, in particular its thickness in comparison with the NIP film. If the film is thicker, its resistance to electron transfer is higher. Also, it is possible that the presence of glyphosate in the MIP film makes it more resistive in comparison with the NIP film (see diagram of the Supplementary Figure 2).

The removal of the pesticide template from the MIPs was lead to significant decrease in impedance (Figure 3A, e) and the formation of two well-defined loops has been observed (for the first semicircle R_2_ = 11,775 Ohm, for the second semicircle R_3_ = 53,728 Ohm). Moreover, charge transfer resistance of NIPs (R_ct_ = 59,890 Ohm) is higher than MIPs film after removing template, due to the presence of imprinted cavities on MIPs layer, which favors the electron transfer.

This result is also proved by the cyclic voltammetry (CV) curves obtained in a ferro-ferricyanide solution (E varying from 0 to 0.45 V and scan rate of 80 mV/s). Like the results of the EIS, those of the CV (Figure 3B) correspond to the same characteristics of the electrodes. It can be seen from the CV (Figure 3B), after electrodeposition of CMA (Figure 3B, c) the decrease of intensity current of redox peaks was recorded compared with bare gold surface (Figure 3B, a). Then, redox peaks is lower after deposition of the CS which is a non-conductor polymer (Figure 3B, d). It was observed that redox peaks of NIPs film (Figure 3B, b) are higher than MIPs film which is thicker. Due to extraction of template from the MIPs matrix (Figure 3B, e), it was observed a significant increase in redox peak, this peak is bigger comparing to NIPs which is related to the presence of imprinted hole promoting the charge transfer.

### Surface Features

**Figure 4:**
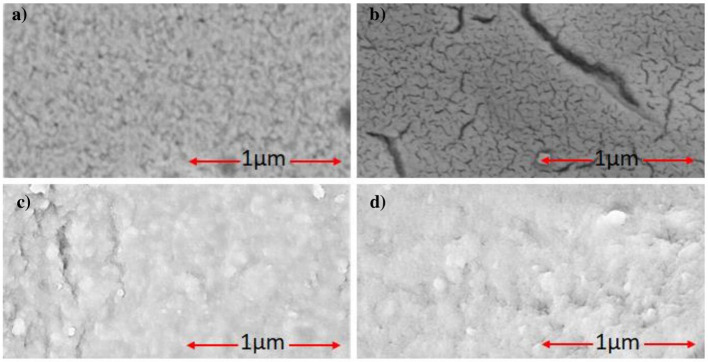
SEM images. **(a)** Bare gold, **(b)** CMA/Au, **(c)** CS-MIPs/CMA/Au, **(d)** CS-NIPs/CMA/Au.

The surface morphologies investigations of CMA/Au, CS-MIPs/CMA/Au and CS-NIP/CMA/Au (Figure 4) were carried out by scanning electron microscopy (SEM). It can be seen from the completely different morphologies of the surfaces of the bare electrodes (Figure 4a) and that of the modified with CMA (Figure 4b), that the operation of depositing the CMA film was well-accomplished. After the chemical deposition of the chitosan onto CMA layer, another morphology modification has appeared on the surface of the microelectrode which confirms the deposition of the CS (Figure 4c). Therefore, SEM does not allow seeing the difference between the morphology of MIPs (Figure 4b) and NIPs films (Figure 4c); because the diameters of the cavities on the chitosan layer have the same order of magnitude with the GLY molecules; in the order of a few nanometers.

### Electrochemical Responses of MIPs to Glyphosate

**Figure 5:**
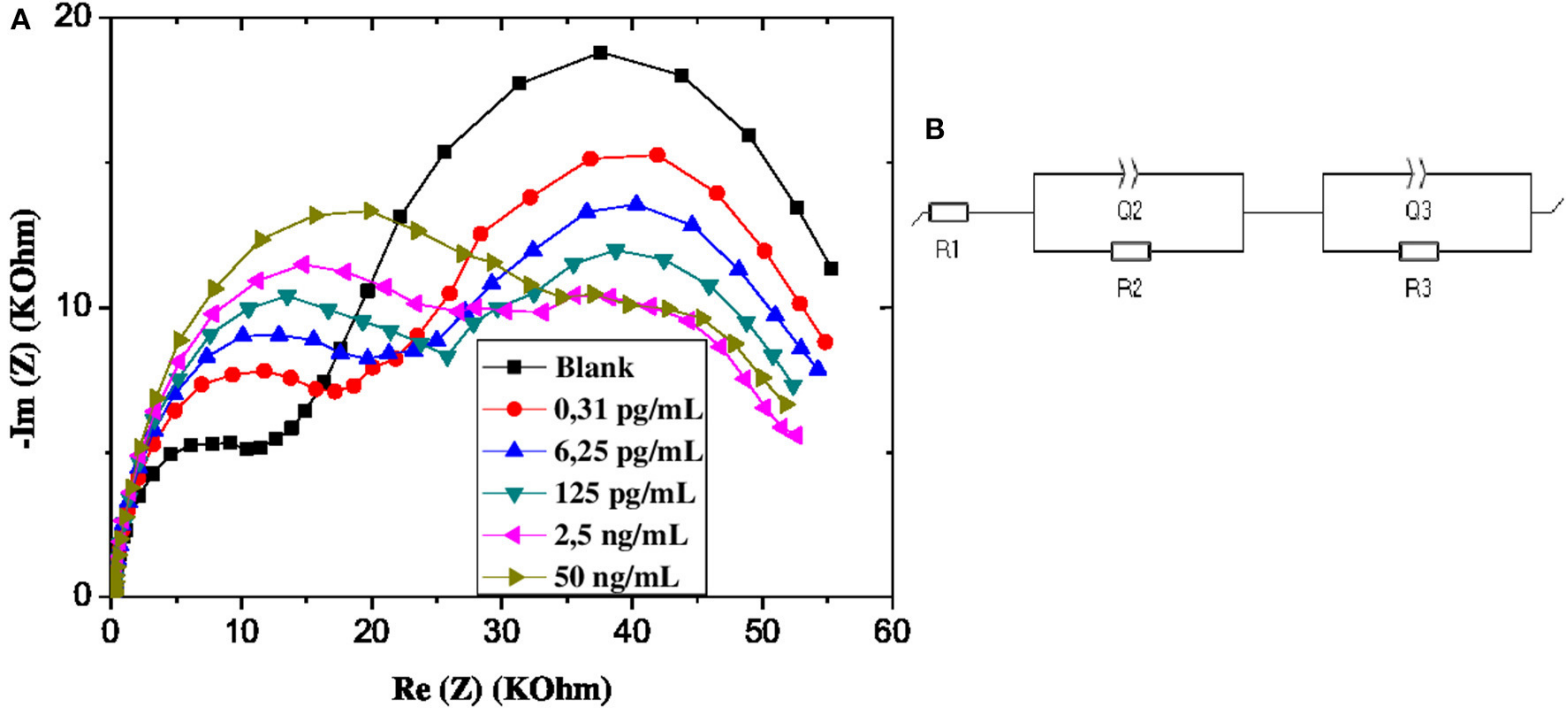
**(A)** EIS of CS-MIPs/CMA/Au in 5 mM [Fe(CN)6]^3−/4−^ and PBS. Before pre-concentration and after incubation in different concentrations of GLY. **(B)** Schematic of equivalent electrical circuit.

From the EIS graphs in Figure 5A, we can see a very remarkable variation in response amplitudes with increasing GLY concentration, which is an indicator of the relationship between the latter and the impedance of the GLY sensor.

The EIS data were fitted by equivalent electrical circuit model (EEC). The optimal fit was done using (R_1_ + Q_2_/R_2_ + Q_3_/R_3_) EEC, which consists of three components associated in series (Figure 5B). The first component R_1_ expresses the electrolyte resistance. The parallel elements Q_2_ and R_2_, which are related to the first Nyquist semi-circle, correspond respectively to the constant phase element (CPE) and the charge transfer resistance. For to the second Nyquist semi-circle, the parallel elements Q_3_ and R_3_, correspond respectively to CPE and charge transfer resistance. This electrical circuit was reported to characterize porous structures. CPE_1_ and CPE_2_ are interpreted as the non-ideal capacitances which are caused by the surface geometry, roughness, and porosity. The relation between these constant phase elements Q and the impedance Z is given by: Z_CPE_ = 1/Q(jω)^n^; where j is the imaginary number, ω is the angular frequency and n is correction factor (0 < n < 1). If the value n tends to 0, CPE becomes less capacitive (Ebdelli et al., 2011; Gaied et al., 2014; Aymen et al., 2015). The selection of the equivalent circuit was dependent upon the fitting of the Nyquist plot to produce the smallest error, expressed as the standard deviation (*X*^2^).

The estimated values of the parameters after adjusting the model of the equivalent circuit with the impedance spectra are given in Table 1. The results reported by the fitting reveals that n_2_ and n_3_ are <1, which confirmed the roughness and the heterogeneity of the microelectrode surface. As these values are invariant with the increase in the applied potential, the stability of the electrode surface is confirmed.

**Table 1:** Estimated values of the parameters fitted by equivalent electrical circuit model R_1_ + Q_2_/R_2_ + Q_3_/R_3_.

**[GLY].10^**9**^ mg/mL**	**R_**1**_ (Ohm)**	**R_**2**_ (Ohm)**	**Q_**2**_. 10^**7**^ (F.s^**n-1**^)**	**n_**2**_**	**R_**3**_ (Ohm)**	**Q_**3**_. 10^**6**^ (F.s^**n-1**^)**	**n_**3**_**	***X*^**2**^**
Blank	147.0	11,775	3.09	0.86	53,728	8.84	0.74	0.042
0.31	142.4	14,633	2.49	0.88	45,421	8.52	0.72	0.013
6.25	135.1	19,369	2.22	0.88	40,029	7.72	0.73	0.014
125	134.8	21,937	2.16	0.88	34,817	7.21	0.73	0.013
2,500	132.6	23,910	2.03	0.87	30,561	6.45	0.73	0.013
50, 000	131.6	28,397	2.83	0.87	28,673	5.73	0.77	0.011

At high frequencies, the Nyquist plot shows a first semi-circle, while the second is observed at lower frequencies. This element is comprised of contributions from three different processes which are electron transfer process, diffusion control and adsorption of ionic species (Terbouche et al., 2016; Tanujit and Asokan, 2019).

The diminution of R_3_ charge transfer resistance is probably related to the adsorption of [Fe(CN)_6_]^3−/4−^ on the CMA microelectrode, or may be attributed to the electrostatic interaction between this negative ions and membranes modified surface. This decrease is due to the accumulation of ions on the surface, which increases the active surface area of the electrode. These behaviors are allowed by the morphology and surface porosity of the CS-MIPs/CMA/Au electrodes.

On another side, the resistance R_2_ increases due to re-occupancy of the complementary sites which are located on the membrane of the biopolymer. These complexations make the surface of the electrode more resistant to charge transfer.

**Figure 6:**
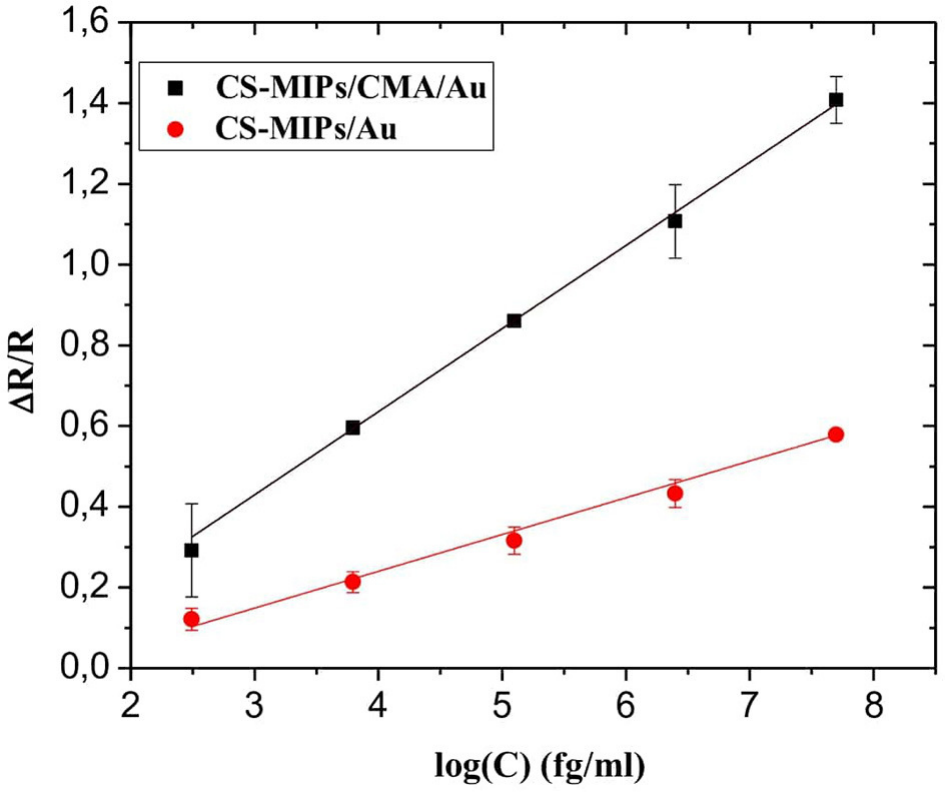
Sensitivity of CS-MIPs/CMA/Au (ΔR/R = 0.206 log [GLY] – 0.188, R^2^ = 0.999) and CS-MIPs/Au (ΔR/R = 0.087 log [GLY] +0.673; R^2^ = 0.991).

If R_2_ and R are the load transfer resistances of the sensor corresponding respectively to a given and zero concentration of GLY, the ratio |R–R_2_|/R_2_ = ΔR/R, therefore makes it possible to measure the relative variation of the charge transfer resistance of the electrode. Therefore, ΔR/R is related to the evolution of the GLY content of the solution. The exploitation of the data of the EIS diagrams made it possible to find a linear relation between this parameter and the logarithm of the concentration of GLY. The linear range is from 0.31 × 10^−9^ up to 50 × 10^−6^ mg/mL as shown in Figure 6. The calibration equation is given below (with a coefficient of determination = 0.998):

ΔR/R=0.206log[GLY]−0.188,

This new sensor has a detection limit (LOD) of 10^−12^ mg/mL. The estimation of the LOD is carried out thanks to the commonly used equation: 3S/m (Shrivastava and Gupta, 2011), S and m are respectively the residual standard deviation and the slope of the calibration line. The high sensitivity of these MIP/Au/CMA sensors allows them to be used successfully for the detection of very low concentrations of GLY. Indeed, the LOD obtained is 1/10^8^ times lower compared to the EQS value of pesticides equal to 0.1 μg/mL.

The analytical performance of the CS-MIPs/CMA/Au sensor was compared to other MIPs electrochemical sensors used for GLY detection reported in the literature (Table 2). To the best of our knowledge, this sensor had the best analytical characteristics than most previously reported sensors.

**Table 2:** A comparison of analytical parameters of the proposed electrode with other MIP electrochemical sensors.

**Electrode**	**Technique**	**Linear range mg/mL**	**Limit of detection mg/mL**	**Sensitivity**	**References**
Ppy-MIP/ HAuCl_4_-PB/ITO	DPV	400 × 10^−6^−1,200 × 10^−6^	92 × 10^−6^	/	Xu et al., 2017
Ppy-MIP/Au	DPV	5 × 10^−6^−800 × 10^−6^	0,27 × 10^−6^	12,8	Zhang et al., 2017
Ppy-MIP/Au	SWV	1,7 × 10^−11^−1,7 × 10^−2^	1,7 × 10^−10^	/	Mazouz et al., 2017
CS-MIP/Au	EIS	0,31 × 10^−9^−50 × 10^−6^	10^−12^	0,087	Zouaoui et al., 2020b
CS-MIP/CMA/Au	EIS	0,31 × 10^−9^−50 × 10^−6^	10^−12^	0,206	This work

*Ppy-MIP/HAuCl_4_-PB/ITO, Molecularly imprinted Polypyrrole/Composite of urchin-like gold nanoparticles and Prussian Blue/Indium-Tin Oxide glass; Ppy-MIP/Au, Molecularly imprinted Polypyrrole/Gold electrode; CS-MIP/Au, Molecularly imprinted Chitosan/Gold microelectrode; DPV, Differential Pulse Voltammetry; SWV, Square Wave Voltammetry*.

### CMA Effect on Sensor Sensitivity

The sensitivity of the sensor based on CS-MIPs modified with CMA (CS-MIPs/CMA/Au) was compared with the sensitivity of the sensor based only on chitosan membrane (CS-MIPs/Au) (Zouaoui et al., 2020b). As shown in Figure 6, the sensitivity of CS-MIPs/CMA/Au is 2.37 times higher than CS-MIPs/Au. For applications in the sensor field, the electrodeposition of chitosan (CS-MIPs/Au) on a conductive surface by pH gradient causes the degassing of H_2_. These molecules are blocked inside the membrane, which makes it non-homogeneous. “The grafting of chitosan onto the diazonium salt by the formation of strong interactions probably leads to more homogeneous membranes and to the resolution of interface problems.”

### Interference

**Figure 7:**
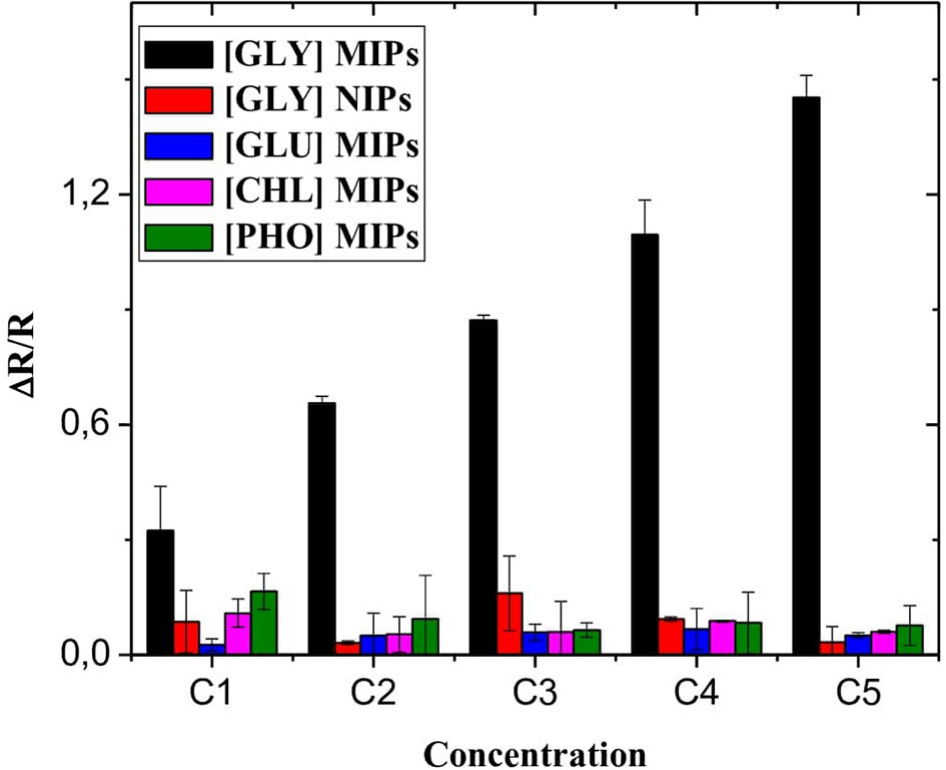
Selectivity of CS-MIPs/CMA/Au microsensor (black) response of CS-MIPs/CMA/Au to GLY, (red) response of CS-NIPs/CMA/Au to GLY, (blue) response of CS-MIPs/CMA/Au to GLU, (pink) response of CS-MIPs/CMA/Au to CHL, and (green) response of CS-MIPs/CMA/Au to PHO.

The selective sensing of the designed microsensor of the GLY was tested using interfering pesticides; this includes phosmet (PHO), gluphosinate-ammonium (GLU), and chlorpyrifos (CHL). EIS analyzes are performed using the same experimental process as that for GLY detection. The detection tests of PHO, GLU, and CHL were performed in the same concentration range between 0.31 × 10^−9^ and 50 × 10^−6^ mg/mL (Figure 7). For the different interferences, the response of CS-MIPs/CMA/Au sensor were lower compared with GLY detection indicating that the microsensor displays the best selectivity to GLY.

Non-imprinted microsensor (CS-NIPs/CMA/Au) was used to study the effectiveness of the imprinting in the presence of GLY. As seen in Figure 7, ΔR/R of the CS-MIPs/CMA/Au were stronger compared with ΔR/R of the CS-NIPs/CMA/Au indicating that the proposed sensor had excellent specificity, because the fixation of GLY molecule by the CS-NIPs/CMA/Au microsensor is very low which confirms the success and efficiency of the GLY's impression.

### About the Sensor Regeneration and Reproducibility

Three working microelectrodes, prepared according to the same method, are used to study the reproducibility of the CS-MIPs/CMA/Au microsensor. Acceptable reproducibility is obtained with a relative standard deviation of 1.46%.

**Figure 8:**
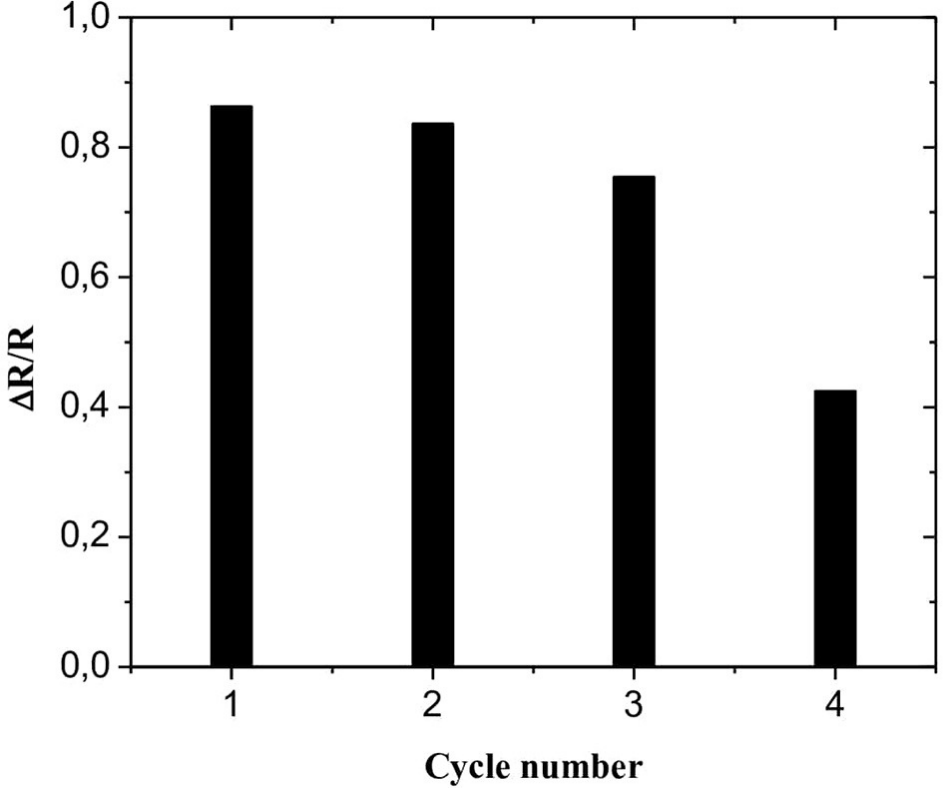
Regeneration of the CS-MIPs/CMA/Au microsensor.

To study the regeneration of the CS-MIPs/CMA/Au microsensor, EIS repetitive measurements were carried out using the same imprinted microsensor which was re-immersed in solutions containing 125 pg/mL of GLY. After each measurement, the CS-MIPs/CMA/Au microsensor was incubated for 5 min in CH_3_COOH/ CH_3_OH solution (1:1, v/v) to eliminate attached GLY molecules. As shown in Figure 8, the results of second and the third repeat measurements showed weak relative variation of ΔR/R compared with the first cycle (3.08 and 12.75%, respectively). For the fourth repeat, the variation was more significant (50.75%), indicating that the sensor can only be regenerated twice.

## Real Sample Analysis

**Figure 9:**
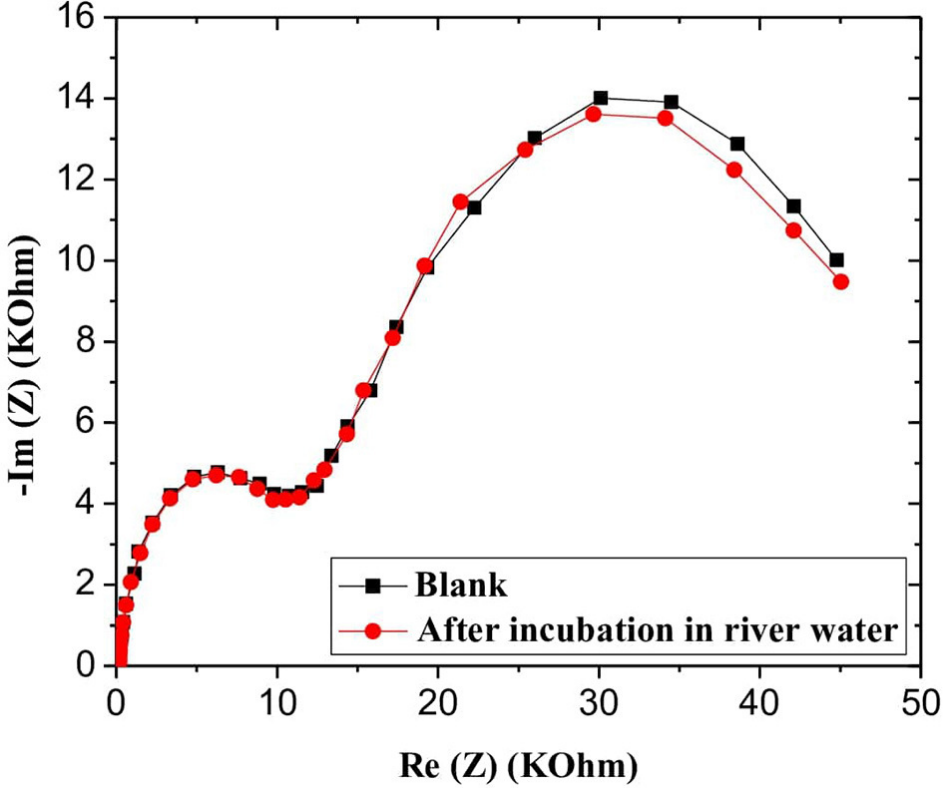
EIS of CS-MIPs/CMA/Au before and after incubation in Rhone river water.

In order to assess the robustness and the applicability of the developed microsensor in a real environment, water samples retrieved from the Rhone River in Lyon, France, were analyzed. CS-MIPs/CMA/Au microsensor was incubated in river water samples for 30 min, and then EIS experiments were carried out for different GLY concentrations. The EIS diagrams were plotted to deduce electron transfer resistance of the sensor surface. Respective Nyquist plots before and after the sample analysis are almost superimposed, probably indicating the absence of GLY in the analyzed sample (Figure 9).

**Figure 10:**
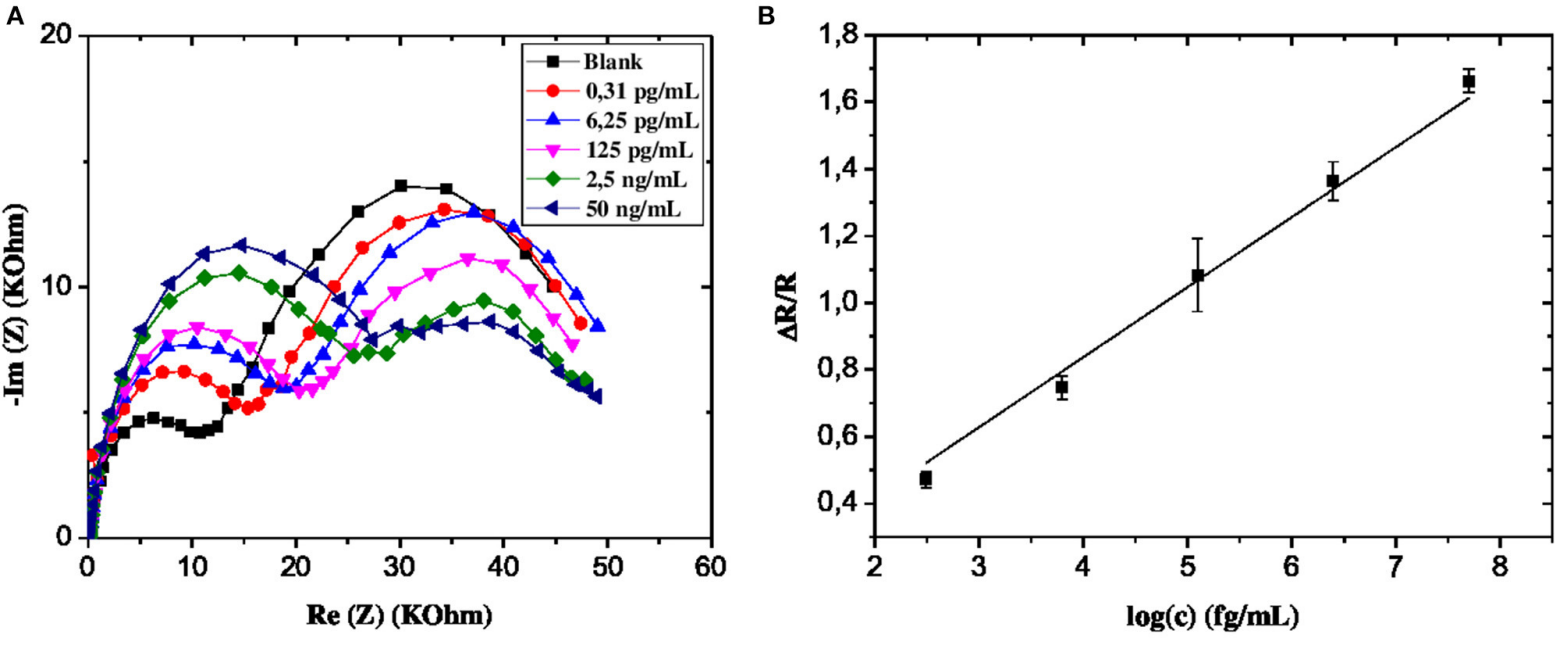
**(A)** EIS of CS-MIPs/CMA/Au in 5 mM [Fe(CN)_6_]^3−/4−^ and PBS Before pre-concentration and after incubation in river water with different concentrations of GLY. **(B)** detection of GLY in Rhone river water using standard addition method with the following concentrations (0.031 pg/mL, 6.25 pg/ml, 125 pg/ml, 2.5 ng/ml, and 50 ng/ml) (ΔR/R = 0.20 log [GLY]; *R*^2^ = 0.997).

To confirm the above observation, the GLY was quantified using the standard additions method. A remarkable variation of impedance spectra was observed with a gradual increase of GLY concentration in the river water sample in the interval of 0.31 × 10^−9^-50 × 10^−6^ mg/mL (Figure 10A). A linear relationship between GLY concentration and the relative transfer resistance has been registered with good correlation coefficient equals to 0.997 (Figure 10B). The form of regression equation y = ax (Δ*R*/*R* = 0.20log[GLY]) is an indication of the absence of GLY in the river water considered. In addition, the sensitivity of this designed microsensor in the real and buffer samples were very close, which indicated that the CS-MIPs/CMA/Au exhibited high reliability.

## Conclusion

This work is a new investigation around the design of a sensitive and selective sensor for the detection of traces of GLY in synthetic and real water samples. It is based on the grafting of the MIP molecularly imprinted CS chitosan onto an Au microelectrode modified with 4-aminophenylacetic acid CMA. Thanks to EIS investigations, the quantification of GLY was carried out to in a linear concentration range between 0.31 × 10^−9^ and 50 × 10^−6^ mg/mL and a limit of detection of 10^−12^ mg/mL.

The sensitivity of CS-MIPs/CMA/Au is higher compared with CS-MIPs/Au which arouses the interest to use this diazonium salt. The developed electrochemical microsensor has very good selectivity for GLY in comparison to three interferent compounds. The printing efficiency was confirmed using non-imprinted microsensor. In addition, the high reliability of this biosensor allows it to be used precisely to detect low traces of GLY in both synthetic and real water samples. The ease of implementation, the simplicity and the miniaturization of this sensor guide research for the development of economical, precise, and rapid tools for environmental analysis.

## Data Availability Statement

The raw data supporting the conclusions of this article will be made available by the authors, without undue reservation.

## Author Contributions

FZ carried out the experimental work under the supervision of NJ-R and AE, and wrote the article. JB and AA contributed to the design of the sensors. NJ-R supervised and contributed writing and editing. MB and SB-B revised and corrected the document. All authors have read and accepted the published version of the manuscript.

## Conflict of Interest

The authors declare that the research was conducted in the absence of any commercial or financial relationships that could be construed as a potential conflict of interest.
