# Supplementary figures and images for: Electrochemical Impedance Spectroscopy Microsensor Based on Molecularly Imprinted Chitosan Film Grafted on a 4-Aminophenylacetic Acid (CMA) Modified Gold Electrode, for the Sensitive Detection of Glyphosate
Source: Front Chem. 2021 May 7;9:621057. doi: 10.3389/fchem.2021.621057 (PMC8145283; doi:10.3389/fchem.2021.621057)

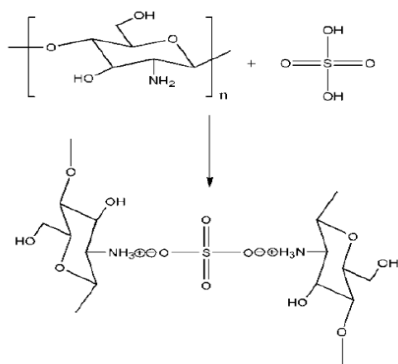

Fig1s: cross linking mechanism of the Cs/H<sub>2</sub>SO<sub>4</sub>

Supplement: Supplementary file 1 [file Data_Sheet_1.PDF]

### NIP film

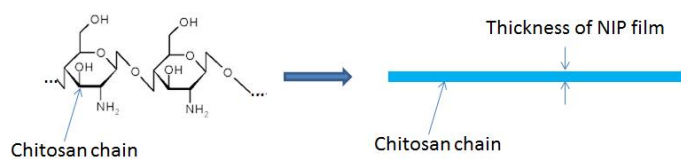

### MIP film

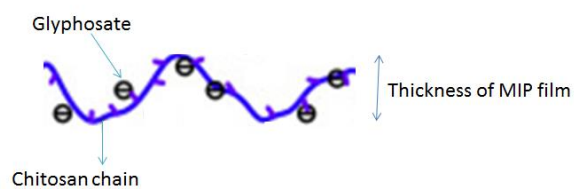

Fig 2s : diagram of the thickness of NIP and MIP films

Supplement: Supplementary file 2 [file Data_Sheet_2.PDF]
